# Supplementary material for: NIgPred: Class-Specific Antibody Prediction for Linear B-Cell Epitopes Based on Heterogeneous Features and Machine-Learning Approaches
Source: Viruses. 2021 Aug 3;13(8):1531. doi: 10.3390/v13081531 (PMC8402714; doi:10.3390/v13081531)
Supplement: Supplementary file 1 [file viruses-13-01531-s001.zip › Supplementary Table S1.pdf]

Supplementary table S1: Significant features by feature selection for IgA, IgE and IgG models

Significant features of IgA model

Rank	Feature type	Function	Detailed feature representation
1	Composition properties	length	Length_train
2	Physicochemical properties	mswhimScores	mswhimScores_train.MSWHIM2
3	Composition properties	extractCTDD	ctd_train.prop4.G2.residue25
4	Composition properties	extractCTDD	ctd_train.prop5.G2.residue50
5	Physicochemical properties	mw	mw
6	Composition properties	extractCTDD	ctd_train.prop2.G1.residue25
7	Peptide characteristic correlation	extractPAAC	pseaac_train.Xc2.lambda.1
8	Composition properties	extractAAC	ac_train.K
9	Composition properties	extractCTDD	ctd_train.prop2.G2.residue0
10	Composition properties	extractCTDD	ctd_train.prop6.G1.residue0
11	Peptide characteristic correlation	extractPAAC	pseaac_train.Xc2.lambda.2
12	Composition properties	extractCTDD	ctd_train.prop4.G2.residue50
13	Composition properties	extractCTDD	ctd_train.prop6.G1.residue75
14	Physicochemical properties	crucianiProperties	crucianiProperties_train.PP3
15	Composition properties	extractCTDD	ctd_train.prop7.G2.residue0

16	Composition properties	extractCTDD	ctd_train.prop1.G1.residue0
17	Composition properties	extractAAC	ac_train.S
18	Composition properties	extractCTDD	ctd_train.prop4.G1.residue0
19	Peptide characteristic correlation	extractPAAC	pseaac_train.Xc2.lambda.3
20	Composition properties	extractDC	dc_train.DE
21	Peptide characteristic correlation	extractPAAC	pseaac_train.Xc2.lambda.4
22	Peptide characteristic correlation	extractPAAC	pseaac_train.Xc1.E
23	Composition properties	extractCTDD	ctd_train.prop4.G2.residue100
24	Composition properties	extractDC	dc_train.KE
25	Physicochemical properties	mswhimScores	mswhimScores_train.MSWHIM1
26	Composition properties	extractAAC	ac_train.T
27	Composition properties	extractAAC	ac_train.N
28	Peptide characteristic correlation	extractAPAAC	APAAC_train.Pc2.Hydrophobicity.1
29	Composition properties	extractDC	dc_train.EK
30	Composition properties	extractCTDD	ctd_train.prop2.G2.residue75
31	Peptide characteristic correlation	extractCTriad	Triad_train.VS422

32	Peptide characteristic correlation	extractPAAC	pseaac_train.Xc1.Q
33	Composition properties	extractAAC	ac_train.R
34	Peptide characteristic correlation	extractCTriad	Triad_train.VS211
35	Composition properties	extractCTDD	ctd_train.prop5.G1.residue50
36	Composition properties	extractAAC	ac_train.C
37	Composition properties	extractCTDD	ctd_train.prop2.G1.residue75
38	Peptide characteristic correlation	extractAPAAC	APAAC_train.Pc1.N
39	Physicochemical properties	blosumIndices	blosumIndices_train.BLOSUM5
40	Composition properties	extractCTDD	ctd_train.prop6.G3.residue25
41	Composition properties	extractDC	dc_train.DK
42	Composition properties	extractCTDD	ctd_train.prop2.G2.residue100
43	Physicochemical properties	blosumIndices	blosumIndices_train.BLOSUM10
44	Peptide characteristic correlation	extractAPAAC	APAAC_train.Pc1.C
45	Peptide characteristic correlation	extractQSO	QSO_train.Grantham.Xr.C
46	Peptide characteristic correlation	extractCTriad	Triad_train.VS113

47	Peptide characteristic correlation	extractCTriad	Triad_train.VS131
48	Peptide characteristic correlation	extractCTriad	Triad_train.VS231
49	Peptide characteristic correlation	extractCTriad	Triad_train.VS322
50	Peptide characteristic correlation	extractCTriad	Triad_train.VS312
51	Peptide characteristic correlation	extractCTriad	Triad_train.VS466
52	Peptide characteristic correlation	extractCTriad	Triad_train.VS242
53	Autocorrelation	extractMoreauBroto	extractMoreauBroto_train.MoreauBroto_feature_11
54	Peptide characteristic correlation	extractCTriad	Triad_train.VS226
55	Peptide characteristic correlation	extractCTriad	Triad_train.VS112
56	Peptide characteristic correlation	extractCTriad	Triad_train.VS661
57	Peptide characteristic correlation	extractCTriad	Triad_train.VS621
58	Autocorrelation	extractMoreauBroto	extractMoreauBroto_train.MoreauBroto_feature_29
59	Autocorrelation	extractMoran	extractMoran_train.Moran_feature_23
60	Autocorrelation	extractMoreauBroto	extractMoreauBroto_train.MoreauBroto_feature_12

61	Peptide characteristic correlation	extractCTriad	Triad_train.VS321
62	Autocorrelation	extractMoreauBroto	extractMoreauBroto_train.MoreauBroto_feature_14
63	Autocorrelation	extractMoran	extractMoran_train.Moran_feature_29
64	Autocorrelation	extractMoreauBroto	extractMoreauBroto_train.MoreauBroto_feature_16
65	Composition properties	extractDC	dc_train.GV
66	Composition properties	extractDC	dc_train.AV
67	Peptide characteristic correlation	extractCTriad	Triad_train.VS236
68	Peptide characteristic correlation	extractCTriad	Triad_train.VS134
69	Peptide characteristic correlation	extractCTriad	Triad_train.VS343
70	Peptide characteristic correlation	extractQSO	QSO_train.Schneider.Xd.2
71	Autocorrelation	extractMoreauBroto	extractMoreauBroto_train.MoreauBroto_feature_15
72	Autocorrelation	extractMoreauBroto	extractMoreauBroto_train.MoreauBroto_feature_22
73	Peptide characteristic correlation	extractCTriad	Triad_train.VS234
74	Peptide characteristic correlation	extractCTriad	Triad_train.VS344
75	Peptide characteristic correlation	extractCTriad	Triad_train.VS323

76	Peptide characteristic correlation	extractCTriad	Triad_train.VS411
77	Peptide characteristic correlation	extractCTriad	Triad_train.VS334
78	Autocorrelation	extractGeary	extractGeary_train.Geary_feature_29
79	Peptide characteristic correlation	extractCTriad	Triad_train.VS512
80	Autocorrelation	extractMoran	extractMoran_train.Moran_feature_8
81	Peptide characteristic correlation	extractCTriad	Triad_train.VS361
82	Peptide characteristic correlation	extractCTriad	Triad_train.VS213
83	Peptide characteristic correlation	extractCTriad	Triad_train.VS342
84	Autocorrelation	extractMoran	extractMoran_train.Moran_feature_27
85	Composition properties	extractDC	dc_train.PS
86	Peptide characteristic correlation	extractCTriad	Triad_train.VS532
87	Autocorrelation	extractMoreauBroto	extractMoreauBroto_train.MoreauBroto_feature_32
88	Peptide characteristic correlation	extractCTriad	Triad_train.VS313
89	Peptide characteristic correlation	extractCTriad	Triad_train.VS256

90	Autocorrelation	extractMoran	extractMoran_train.Moran_feature_24
91	Peptide characteristic correlation	extractCTriad	Triad_train.VS362
92	Peptide characteristic correlation	extractCTriad	Triad_train.VS435
93	Peptide characteristic correlation	extractCTriad	Triad_train.VS144
94	Peptide characteristic correlation	extractCTriad	Triad_train.VS463
95	Peptide characteristic correlation	extractCTriad	Triad_train.VS433
96	Peptide characteristic correlation	extractCTriad	Triad_train.VS316
97	Peptide characteristic correlation	extractCTriad	Triad_train.VS225
98	Peptide characteristic correlation	extractCTriad	Triad_train.VS243
99	Peptide characteristic correlation	extractCTriad	Triad_train.VS613
100	Peptide characteristic correlation	extractCTriad	Triad_train.VS262
101	Peptide characteristic correlation	extractCTriad	Triad_train.VS524
102	Peptide characteristic correlation	extractCTriad	Triad_train.VS331

103	Peptide characteristic correlation	extractCTriad	Triad_train.VS446
104	Peptide characteristic correlation	extractCTriad	Triad_train.VS141
105	Composition properties	extractDC	dc_train.RT
106	Peptide characteristic correlation	extractCTriad	Triad_train.VS124
107	Peptide characteristic correlation	extractCTriad	Triad_train.VS441
108	Autocorrelation	extractMoreauBroto	extractMoreauBroto_train.MoreauBroto_feature_30
109	Peptide characteristic correlation	extractCTriad	Triad_train.VS163
110	Peptide characteristic correlation	extractCTriad	Triad_train.VS241
111	Peptide characteristic correlation	extractCTriad	Triad_train.VS133
112	Composition properties	extractAAC	ac_train.W
113	Peptide characteristic correlation	extractCTriad	Triad_train.VS542
114	Peptide characteristic correlation	extractCTriad	Triad_train.VS662
115	Composition properties	extractDC	dc_train.GR
116	Autocorrelation	extractGeary	extractGeary_train.Geary_feature_19

117	Autocorrelation	extractGeary	extractGeary_train.Geary_feature_23
118	Peptide characteristic correlation	extractCTriad	Triad_train.VS245

Significant features of IgE model

Rank	Feature type	Function	Detailed feature representation
1	Physicochemical properties	crucianiProperties	crucianiProperties_train.PP1
2	Composition properties	extractCTDD	ctd_train.prop3.G3.residue100
3	Composition properties	extractCTDD	ctd_train.prop7.G2.residue75
4	Composition properties	extractCTDD	ctd_train.prop1.G1.residue75
5	Composition properties	extractCTDD	ctd_train.prop7.G2.residue100
6	Composition properties	extractCTDD	ctd_train.prop1.G1.residue100
7	Physicochemical properties	aacomp	aaComp_train.NonPolar
8	Composition properties	extractCTDD	ctd_train.prop7.G2.residue50
9	Composition properties	extractCTDD	ctd_train.prop1.G1.residue50
10	Physicochemical properties	boman	boman
11	Composition properties	extractCTDD	ctd_train.prop3.G3.residue50
12	Composition properties	extractCTDD	ctd_train.prop3.G2.residue0
13	Composition properties	extractCTDD	ctd_train.prop1.G2.residue0
14	Composition properties	extractCTDD	ctd_train.prop5.G2.residue0

15	Composition properties	extractCTDD	ctd_train.prop1.G2.residue25
16	Composition properties	extractCTDD	ctd_train.prop5.G1.residue100
17	Composition properties	extractCTDD	ctd_train.prop7.G1.residue25
18	Composition properties	extractAAC	ac_train.V
19	Physicochemical properties	Zscales	zScales_train.Z1
20	Composition properties	extractCTDD	ctd_train.prop3.G1.residue0
21	Composition properties	extractCTDD	ctd_train.prop1.G3.residue0
22	Composition properties	extractCTDD	ctd_train.prop2.G3.residue50
23	Composition properties	extractCTDD	ctd_train.prop4.G3.residue50
24	Composition properties	extractCTDD	ctd_train.prop1.G2.residue75
25	Composition properties	extractCTDD	ctd_train.prop4.G1.residue25
26	Composition properties	extractCTDD	ctd_train.prop1.G2.residue50
27	Composition properties	extractCTDD	ctd_train.prop7.G3.residue75
28	Composition properties	extractCTDD	ctd_train.prop3.G1.residue25
29	Composition properties	extractCTDD	ctd_train.prop2.G2.residue25
30	Composition properties	extractCTDD	ctd_train.prop6.G1.residue25
31	Composition properties	extractCTDD	ctd_train.prop5.G3.residue75
32	Composition properties	extractCTDD	ctd_train.prop7.G3.residue25
33	Composition properties	extractCTDD	ctd_train.prop2.G1.residue50

34	Composition properties	extractAAC	ac_train.P
35	Composition properties	extractCTDD	ctd_train.prop7.G1.residue75
36	Peptide characteristic correlation	extractAPAAC	APAAC_train.Pc2.Hydrophobicity.1
37	Composition properties	extractCTDD	ctd_train.prop6.G2.residue0
38	Physicochemical properties	aacomp	aaComp_train.Small
39	Physicochemical properties	blosumIndices	blosumIndices_train.BLOSUM7
40	Peptide characteristic correlation	extractCTriad	Triad_train.VS223
41	Composition properties	extractCTDD	ctd_train.prop3.G2.residue50
42	Peptide characteristic correlation	fasgaiVectors	fasgaiVectors_train.F1
43	Composition properties	extractCTDD	ctd_train.prop6.G2.residue25
44	Physicochemical properties	aindex	aIndex
45	Composition properties	extractAAC	ac_train.K
46	Composition properties	extractCTDD	ctd_train.prop5.G1.residue0
47	Composition properties	extractAAC	ac_train.T
48	Composition properties	extractCTDD	ctd_train.prop6.G3.residue75
49	Composition properties	extractCTDD	ctd_train.prop5.G3.residue100
50	Composition properties	extractCTDD	ctd_train.prop2.G1.residue75

51	Composition properties	extractCTDD	ctd_train.prop4.G3.residue75
52	Composition properties	extractCTDD	ctd_train.prop2.G3.residue75
53	Composition properties	extractCTDD	ctd_train.prop2.G2.residue75
54	Composition properties	extractCTDD	ctd_train.prop7.G1.residue0
55	Composition properties	extractCTDD	ctd_train.prop5.G3.residue0
56	Composition properties	extractCTDD	ctd_train.prop2.G3.residue0
57	Composition properties	extractAAC	ac_train.S
58	Composition properties	extractCTDD	ctd_train.prop6.G3.residue0
59	Physicochemical properties	aacomp	aaComp_train.Charged
60	Composition properties	extractCTDD	ctd_train.prop2.G2.residue100
61	Composition properties	extractCTDD	ctd_train.prop2.G3.residue25
62	Peptide characteristic correlation	extractAPAAC	APAAC_train.Pc2.Hydrophobicity.2
63	Composition properties	extractCTDD	ctd_train.prop1.G1.residue25
64	Composition properties	extractCTDD	ctd_train.prop2.G3.residue100
65	Composition properties	extractCTDD	ctd_train.prop4.G3.residue100
66	Composition properties	extractDC	dc_train.AV
67	Composition properties	extractCTDD	ctd_train.prop5.G3.residue25
68	Composition properties	extractCTDD	ctd_train.prop4.G2.residue75

69	Peptide characteristic correlation	extractCTriad	Triad_train.VS121
70	Peptide characteristic correlation	extractAPAAC	APAAC_train.Pc1.A
71	Peptide characteristic correlation	extractPAAC	pseaac_train.Xc1.T
72	Physicochemical properties	aacomp	aaComp_train.Acidic
73	Autocorrelation	extractMoreau Broto	extractMoreauBroto_train.Moreau Broto_feature_9
74	Peptide characteristic correlation	extractAPAAC	APAAC_train.Pc2.Hydrophilicity.3
75	Peptide characteristic correlation	extractAPAAC	APAAC_train.Pc2.Hydrophilicity.4
76	Composition properties	extractCTDD	ctd_train.prop6.G3.residue100
77	Composition properties	extractCTDD	ctd_train.prop7.G1.residue50
78	Composition properties	extractCTDD	ctd_train.prop1.G3.residue100
79	Peptide characteristic correlation	extractAPAAC	APAAC_train.Pc1.P
80	Physicochemical properties	aacomp	aaComp_train.Tiny
81	Composition properties	extractCTDD	ctd_train.prop3.G3.residue25
82	Peptide characteristic correlation	extractQSO	QSO_train.Grantham.Xr.L
83	Physicochemical properties	crucianiProper ties	crucianiProperties_train.PP2

84	Peptide characteristic correlation	extractAPAAC	APAAC_train.Pc2.Hydrophobicity.4
85	Composition properties	extractCTDD	ctd_train.prop7.G1.residue100
86	Peptide characteristic correlation	extractAPAAC	APAAC_train.Pc2.Hydrophobicity.3
87	Composition properties	extractCTDD	ctd_train.prop6.G3.residue50
88	Autocorrelation	extractMoran	extractMoran_train.Morafeature_4
89	Peptide characteristic correlation	extractAPAAC	APAAC_train.Pc1.K
90	Composition properties	extractAAC	ac_train.Q
91	Composition properties	extractCTDD	ctd_train.prop5.G2.residue100
92	Peptide characteristic correlation	extractQSO	QSO_train.Grantham.Xr.E
93	Composition properties	extractCTDD	ctd_train.prop6.G2.residue75
94	Peptide characteristic correlation	extractAPAAC	APAAC_train.Pc1.R
95	Composition properties	extractCTDD	ctd_train.prop2.G1.residue100
96	Peptide characteristic correlation	extractCTriad	Triad_train.VS612
97	Autocorrelation	extractMoran	extractMoran_train.Moran_feature_32
98	Peptide characteristic correlation	extractCTriad	Triad_train.VS432

99	Peptide characteristic correlation	extractCTriad	Triad_train.VS325
100	Composition properties	extractAAC	ac_train.M
101	Composition properties	extractCTDD	ctd_train.prop4.G1.residue75
102	Autocorrelation	extractMoran	extractMoran_train.Moran_feature_10
103	Physicochemical properties	fasgaiVectors	fasgaiVectors_train.F5
104	Peptide characteristic correlation	extractQSO	QSO_train.Schneider.Xr.N
105	Peptide characteristic correlation	extractCTriad	Triad_train.VS251
106	Peptide characteristic correlation	extractAPAAC	APAAC_train.Pc1.H
107	Autocorrelation	extractMoran	extractMoran_train.Moran_feature_29
108	Composition properties	extractAAC	ac_train.W
109	Autocorrelation	extractGeary	extractGeary_train.Geary_feature_16
110	Physicochemical properties	blosumIndices	blosumIndices_train.BLOSUM6
111	Autocorrelation	extractMoreauBroto	extractMoreauBroto_train.MoreauBroto_feature_29
112	Peptide characteristic correlation	extractCTriad	Triad_train.VS335
113	Peptide characteristic correlation	extractAPAAC	APAAC_train.Pc1.Q
114	Composition properties	extractDC	dc_train.SV

115	Composition properties	extractDC	dc_train.HT
116	Peptide characteristic correlation	extractCTriad	Triad_train.VS641
117	Autocorrelation	extractMoreauBroto	extractMoreauBroto_train.MoreauBroto_feature_31
118	Peptide characteristic correlation	extractAPAAC	APAAC_train.Pc1.F
119	Composition properties	extractCTDD	ctd_train.prop7.G3.residue100
120	Peptide characteristic correlation	extractCTriad	Triad_train.VS266
121	Peptide characteristic correlation	extractCTriad	Triad_train.VS245
122	Autocorrelation	extractGeary	extractGeary_train.Geary_feature_29
123	Peptide characteristic correlation	extractAPAAC	APAAC_train.Pc1.W
124	Peptide characteristic correlation	extractQSO	QSO_train.Grantham.Xr.F
125	Peptide characteristic correlation	extractCTriad	Triad_train.VS632
126	Composition properties	extractDC	dc_train.AS
127	Peptide characteristic correlation	extractCTriad	Triad_train.VS633
128	Composition properties	extractDC	dc_train.TG

129	Peptide characteristic correlation	extractCTriad	Triad_train.VS636
130	Peptide characteristic correlation	extractAPAAC	APAAC_train.Pc1.S
131	Composition properties	extractDC	dc_train.KY
132	Peptide characteristic correlation	extractCTriad	Triad_train.VS621
133	Composition properties	extractDC	dc_train.EA
134	Peptide characteristic correlation	extractCTriad	Triad_train.VS154
135	Peptide characteristic correlation	extractCTriad	Triad_train.VS644
136	Composition properties	extractDC	ac_train.F
137	Peptide characteristic correlation	extractCTriad	Triad_train.VS424
138	Composition properties	extractDC	dc_train.AI
139	Composition properties	extractDC	dc_train.CK
140	Composition properties	extractDC	dc_train.CD
141	Peptide characteristic correlation	extractCTriad	Triad_train.VS463
142	Peptide characteristic correlation	extractCTriad	Triad_train.VS361
143	Composition properties	extractDC	dc_train.SN

144	Composition properties	extractDC	dc_train.SF
145	Composition properties	extractDC	dc_train.QC
146	Composition properties	extractDC	dc_train.DA
147	Composition properties	extractDC	dc_train.FN
148	Composition properties	extractDC	dc_train.NA
149	Peptide characteristic correlation	extractCTriad	Triad_train.VS436
150	Peptide characteristic correlation	extractCTriad	Triad_train.VS742
151	Composition properties	extractDC	dc_train.DK
152	Composition properties	extractDC	dc_train.AY
153	Composition properties	extractDC	dc_train.DN
154	Composition properties	extractDC	dc_train.IR
155	Composition properties	extractDC	dc_train.IT
156	Composition properties	extractDC	dc_train.YG
157	Composition properties	extractDC	dc_train.YN
158	Composition properties	extractDC	dc_train.HP
159	Peptide characteristic correlation	extractCTriad	Triad_train.VS713
160	Peptide characteristic correlation	extractCTriad	Triad_train.VS673

161	Composition properties	extractDC	dc_train.YK
162	Peptide characteristic correlation	extractCTriad	Triad_train.VS774
163	Peptide characteristic correlation	extractCTriad	Triad_train.VS374
164	Peptide characteristic correlation	extractCTriad	Triad_train.VS373
165	Composition properties	extractDC	dc_train.EP
166	Composition properties	extractDC	dc_train.GT
167	Peptide characteristic correlation	extractCTriad	Triad_train.VS157
168	Composition properties	extractDC	dc_train.IC
169	Peptide characteristic correlation	extractCTriad	Triad_train.VS743
170	Peptide characteristic correlation	extractCTriad	Triad_train.VS237
171	Peptide characteristic correlation	extractCTriad	Triad_train.VS724
172	Physicochemical properties	blosumIndices	blosumIndices_train.BLOSUM8
173	Composition properties	extractDC	dc_train.SS
174	Composition properties	extractDC	dc_train.YS
175	Peptide characteristic correlation	extractCTriad	Triad_train.VS277

176	Autocorrelation	extractGeary	extractGeary_train.Geary_feature_13
177	Composition properties	extractDC	dc_train.PG
178	Composition properties	extractDC	dc_train.FE
179	Composition properties	extractDC	dc_train.WQ
180	Composition properties	extractDC	dc_train.HF
181	Peptide characteristic correlation	extractCTriad	Triad_train.VS672
182	Peptide characteristic correlation	extractAPAAC	APAAC_train.Pc1.T
183	Composition properties	extractDC	dc_train.DM

Significant features of IgG model

Rank	Feature type	Function	Detailed feature representation
1	Composition properties	length	Length_train
2	Peptide characteristic correlation	extractAPAAC	APAAC_train.Pc1.W
3	Peptide characteristic correlation	extractCTriad	Triad_train.VS113
4	Peptide characteristic correlation	extractCTriad	Triad_train.VS122
5	Peptide characteristic correlation	extractCTriad	Triad_train.VS322
6	Peptide characteristic correlation	extractCTriad	Triad_train.VS123
7	Peptide characteristic correlation	extractCTriad	Triad_train.VS331
8	Peptide characteristic correlation	extractCTriad	Triad_train.VS611
9	Peptide characteristic correlation	extractCTriad	Triad_train.VS321
10	Peptide characteristic correlation	extractCTriad	Triad_train.VS121
11	Peptide characteristic correlation	extractCTriad	Triad_train.VS114
12	Peptide characteristic correlation	extractCTriad	Triad_train.VS124

13	Peptide characteristic correlation	extractCTriad	Triad_train.VS423
14	Peptide characteristic correlation	extractCTriad	Triad_train.VS323
15	Peptide characteristic correlation	extractCTriad	Triad_train.VS334
16	Peptide characteristic correlation	extractCTriad	Triad_train.VS431
17	Peptide characteristic correlation	extractCTriad	Triad_train.VS422
18	Peptide characteristic correlation	extractCTriad	Triad_train.VS413
19	Peptide characteristic correlation	extractCTriad	Triad_train.VS613
20	Peptide characteristic correlation	extractCTriad	Triad_train.VS324
21	Peptide characteristic correlation	extractCTriad	Triad_train.VS421
22	Peptide characteristic correlation	extractCTriad	Triad_train.VS163
23	Peptide characteristic correlation	extractCTriad	Triad_train.VS622
24	Peptide characteristic correlation	extractCTriad	Triad_train.VS522

25	Peptide characteristic correlation	extractCTriad	Triad_train.VS166
26	Peptide characteristic correlation	extractCTriad	Triad_train.VS621
27	Peptide characteristic correlation	extractCTriad	Triad_train.VS424
28	Peptide characteristic correlation	extractCTriad	Triad_train.VS126
29	Peptide characteristic correlation	extractCTriad	Triad_train.VS253
30	Peptide characteristic correlation	extractCTriad	Triad_train.VS351
31	Peptide characteristic correlation	extractCTriad	Triad_train.VS336
32	Peptide characteristic correlation	extractCTriad	Triad_train.VS523
33	Peptide characteristic correlation	extractCTriad	Triad_train.VS326
34	Peptide characteristic correlation	extractCTriad	Triad_train.VS615
35	Peptide characteristic correlation	extractCTriad	Triad_train.VS251
36	Peptide characteristic correlation	extractCTriad	Triad_train.VS623

37	Peptide characteristic correlation	extractCTriad	Triad_train.VS614
38	Peptide characteristic correlation	extractCTriad	Triad_train.VS521
39	Peptide characteristic correlation	extractCTriad	Triad_train.VS414
40	Peptide characteristic correlation	extractCTriad	Triad_train.VS353
41	Peptide characteristic correlation	extractCTriad	Triad_train.VS164
42	Peptide characteristic correlation	extractCTriad	Triad_train.VS624
43	Peptide characteristic correlation	extractCTriad	Triad_train.VS165
44	Peptide characteristic correlation	extractCTriad	Triad_train.VS254
45	Peptide characteristic correlation	extractCTriad	Triad_train.VS451
46	Peptide characteristic correlation	extractCTriad	Triad_train.VS354
47	Peptide characteristic correlation	extractCTriad	Triad_train.VS415
48	Peptide characteristic correlation	extractCTriad	Triad_train.VS436

49	Peptide characteristic correlation	extractCTriad	Triad_train.VS434
50	Peptide characteristic correlation	extractCTriad	Triad_train.VS356
51	Peptide characteristic correlation	extractCTriad	Triad_train.VS453
52	Peptide characteristic correlation	extractCTriad	Triad_train.VS524
53	Peptide characteristic correlation	extractCTriad	Triad_train.VS463
54	Peptide characteristic correlation	extractCTriad	Triad_train.VS256
55	Peptide characteristic correlation	extractCTriad	Triad_train.VS355
56	Peptide characteristic correlation	extractCTriad	Triad_train.VS464
57	Peptide characteristic correlation	extractCTriad	Triad_train.VS465
58	Peptide characteristic correlation	extractCTriad	Triad_train.VS172
59	Peptide characteristic correlation	extractCTriad	Triad_train.VS173
60	Peptide characteristic correlation	extractCTriad	Triad_train.VS327

61	Peptide characteristic correlation	extractCTriad	Triad_train.VS257
62	Peptide characteristic correlation	extractCTriad	Triad_train.VS427
63	Peptide characteristic correlation	extractCTriad	Triad_train.VS167
