# Supplementary material for: NIgPred: Class-Specific Antibody Prediction for Linear B-Cell Epitopes Based on Heterogeneous Features and Machine-Learning Approaches
Source: Viruses. 2021 Aug 3;13(8):1531. doi: 10.3390/v13081531 (PMC8402714; doi:10.3390/v13081531)
Supplement: Supplementary file 1 [file viruses-13-01531-s001.zip › Supplymentary File S1.pdf]

Supplementary file S1

Amino acid composition

extractAAC: The fragment of each amino acid class inside a protein sequence.

$$f(i)= \frac{N_i}{N}$$

N_i is the amino acid class i and N is the length of the protein sequence

extractDC: The composition of dipeptide class inside a protein sequence.

$$f(i,j)= \frac{N_{ij}}{N}$$

N_{ij} is the dipeptide class i and j , N is the length of the protein sequence

extractCTDD: The distribution of each amino acid attribute in the protein sequence.
Step 1: encoded the protein sequence according to hydrophobicity, normalized van der Waals volume, polarity, and polarizability into three groups.
Step 2: Calculate the first , 25% ,50%,75%,100% residues in whole protein sequence.

Length: Protein sequence of length.

Physicochemical properties

aindex: Calculate the realative volume occupied by aliphatic side(alanine, valine, isoleucine, and leucine).
boman : Calculate the potential protein interaction index according to Boman(2003) into protein sequence.
mw: Calculate sum of mass to each amino acid using scale available on Compute pl/Mw tool.
crucianiProperties: Calculate interaction of amino acid with several chemical group(polartity, H-bonding…) using scaled principal component scores.
hydrophobicity: Calculate the GRAVY hydrophobicity index of a protein sequence using KyteDoolittle scale.
aacomp: Calculate the percentage of tiny, small, aliphatic, aromatic, non-polar, polar, charged, basic and acidic property of protein sequence.
blosumIndices: Calculate the physicochemical properties via VARUMAX analyses and using BLOSUM62 matrix of protein sequence.
Pi: Calculate the isoelectric point of the protein sequence.

mswhimScores: Calculate the MS-WHIM score(36 electrostatic properties) from three dimensional structure of protein sequence.
kideraFactors: Calculate amino acid to 188 physical properties(Helix/bend, Side-chain size…) via multivariate analysis and using dimension reduction algorithm.
fasgaiVectors: Calculate amino acid properties via factor analysis scales of reflects hydrophobicity, alpha and turn, bulky, compositional characteristics, local flexibility, electronic , using above feautre to represent the protein sequence structure features.
vhseScales: Using principal components analysis(PCA) of 18 hydrophobic properties, 17 steric properties, and 15 electronic properties to protein sequence.
Zscales: Calculate average of z-scales amino acid physicochemical properties(Lipophilicity, Steric properties, Electronic properties ,heat of formation, hardness) from NMR data and thin-layer chromatography (TLC) data .

Autocorrelation

Autocorrelation denotes a molecular distribution of amino acid of sequence, using centralized and standardized AAindex database of normalized average hydrophobicity scales, average flexibility Indices, polarizability parameter, free energy of solution in water, kcal/mole, residue accessible surface area in tripeptide, residue volume, steric parameter, relative mutability to compute each amino acid properties.

extractMoreauBroto:

The moreau-broto autocorrelation can be defined as:

$$MB(j) = \sum_{i=1}^{N-j} P_i \, P_{i+j} \; j = 1,2 \ldots, nlag$$

j is called lag of autocorrelation, P_i, P_{i+j} are the properties of amino acid at position i and $i + j$, nlag is the maximum value of lag, we set nlag equal 5 and normailzed moreau-broto autocorrelation is defines as:

$$\mathrm{NMB(j)} = \frac{MB(j)}{N-j} \; j = 1,2 \ldots, nlag$$

extractMoran:

The moran autocorrelation descriptors is define as:

$$\mathrm{M(j)} = \frac{\frac{1}{N-j}\sum_{i=1}^{N-j}(p_i - \bar{p}')(P_{i+j} - \bar{p}')}{\frac{1}{N}\sum_{i=1}^{N}(p_i - \bar{p}')^2} \; j = 1,2\ldots,\mathrm{nlag}$$

$\bar{p}'$ is the considered property P along the sequence,

$$\bar{p}' = \frac{\sum_{i=1}^{N} P_i}{N}$$

extractGeary:

The geary autocorrelation descriptors is define as:

$$G(j) = \frac{\frac{1}{2(N-j)}\sum_{i=1}^{N-j}(p_i - P_{i+j})^2}{\frac{1}{N-1}\sum_{i=1}^{N}(p_i - \bar{p}\prime)^2} \quad j = 1,2\ldots,nlag$$

Protein distance features

The sequence oreder coupling number and Quasi-sequence-order descriptors are purpose by Chou(2000) to compute 20 amino acid distance from Schneider-Wrede physicochemical distance matrix (Schneider and Wrede, 1994) and chemical distance matrix by Grantham (1974).

extractSOCN: The sequence oreder coupling number is defines as:

$$T(j) = \sum_{i=1}^{N-j}(j_{i,i+j})^2$$

$j_{i,i+j}$ is the two amino acid between distance at position i and $i + j$.

extractQSO: The first 20 quasi-sequnce order is defines as:

$$X_r = \frac{f_r}{\sum_{r=1}^{20} fr + w\sum_{j=1}^{maxlag} T_j} \quad r = 1,2\ldots.,20$$

The other 30 quasi-sequnce order is define as

$$X_d = \frac{WT_{j-20}}{\sum_{r=1}^{20} fr + w\sum_{j=1}^{maxlag} T_d} \quad r = 21,22\ldots.,maxlag$$

f_r is the normalized occurrence for amino acid type i and w is weight factor(w =0.1).

extractPAAC and extractAPAAC: The pseudo amino acid composition and amphiphilic pseudo amino acid composition are purpose in 2001 years and 2005 years by Chou, These features are author try to represent long protein sequences order pattern. pseudo amino acid composition descriptors are calculate correlation with 20 natural amino acids of original hydrophobicity values, the original hydrophilicity values and the original side chain masses and amphiphilic pseudo amino acid composition descriptors are calculate correlation with 20 natural amino acids of original hydrophobicity values, the original hydrophilicity values.

extractCTriad: Conjoint triad descriptors are used electrostatic and hydrophobic domain protein protein interaction to classification amino acids. Based on electrostatic and hydrophobic can be classify into seven class and compute per three amino acid freqnce to present amino acid correlation.

References

P. R.-V. Daniel Osorio, R. Torres, Calculate indices and theoretical properties of protein sequences. URL https://github.com/dosorio/Peptides/.

Nan Xiao, Dong-Sheng Cao, Min-Feng Zhu, and Qing-Song Xu. (2015). protr/ProtrWeb: R package and web server for generating various numerical representation schemes of protein sequences. *Bioinformatics* 31 (11), 1857-1859.

Boman, H. G. (2003). Antibacterial peptides: basic facts and emerging concepts. Journal of Internal Medicine, 254(3), 197-215.

Gasteiger, E., Hoogland, C., Gattiker, A., Wilkins, M. R., Appel, R. D., & Bairoch, A. (2005). Protein identification and analysis tools on the ExPASy server. In The proteomics protocols handbook (pp. 571-607). Humana Press. Chicago

Cruciani, G., Baroni, M., Carosati, E., Clementi, M., Valigi, R., and Clementi, S. (2004) Peptide studies by means of principal properties of amino acids derived from MIF descriptors. J. Chemom. 18, 146-155.

Kyte J., Doolittle R.F. Hydropathicity. J. Mol. Biol. 157:105-132(1982).

Rice, Peter, Ian Longden, and Alan Bleasby. "EMBOSS: the European molecular biology open software suite." Trends in genetics 16.6 (2000): 276-277.

Georgiev, A. G. (2009). Interpretable numerical descriptors of amino acid space. Journal of Computational Biology, 16(5), 703-723.

Zaliani, A., & Gancia, E. (1999). MS-WHIM scores for amino acids: a new 3D-description for peptide QSAR and QSPR studies. Journal of chemical information and computer sciences, 39(3), 525-533.

Kidera, A., Konishi, Y., Oka, M., Ooi, T., & Scheraga, H. A. (1985). Statistical analysis of the physical properties of the 20 naturally occurring amino acids. Journal of Protein Chemistry, 4(1), 23-55.

Liang, G., & Li, Z. (2007). Factor analysis scale of generalized amino acid information as the source of a new set of descriptors for elucidating the structure and activity relationships of cationic antimicrobial peptides. Molecular Informatics, 26(6), 754-763.

Mei, H. U., Liao, Z. H., Zhou, Y., & Li, S. Z. (2005). A new set of amino acid descriptors and its application in peptide QSARs. Peptide Science, 80(6), 775-786.

Sandberg M, Eriksson L, Jonsson J, Sjostrom M, Wold S: New chemical descriptors relevant for the design of biologically active peptides. A multivariate characterization of 87 amino acids. J Med Chem 1998, 41:2481-2491.

Feng, Z.P. and Zhang, C.T. (2000) Prediction of membrane protein types based on the hydrophobic index of amino acids. Journal of Protein Chemistry, 19, 269-275.

Horne, D.S. (1988) Prediction of protein helix content from an autocorrelation analysis of sequence hydrophobicities. Biopolymers, 27, 451-477.

Sokal, R.R. and Thomson, B.A. (2006) Population structure inferred by local spatial autocorrelation: an usage from an Amerindian tribal population. American Journal of Physical Anthropology, 129, 121-131.

Kuo-Chen Chou. Prediction of Protein Subcellar Locations by Incorporating Quasi-Sequence-Order Effect. Biochemical and Biophysical Research Communications, 2000, 278, 477-483.

Chou,K.-C. (2000) Prediction of protein subcellar locations by incorporating quasi-sequence-order effect. Biochemical and Biophysical Research Communications, 278, 477–483.

Chou,K.-C. (2005) Using amphiphilic pseudo amino acid composition to predict enzyme subfamily classes. Bioinformatics, 21, 10–19.

Chou,K.-C. and Shen,H.-B. (2008) Cell-ploc: A package of web servers for predicting subcellular localization of proteins in various organisms. Nature Protocols, 3, 153–162.

Shen,J.W. et al. (2007) Predicting protein-protein interactions based only on sequences information. Proceedings of the National Academy of Sciences, 104, 4337–4341.
